# Sociality and parasite transmission

**DOI:** 10.1007/s00265-021-03092-3

**Published:** 2021-10-23

**Authors:** Paul Schmid-Hempel

**Affiliations:** Institute of Integrative Biology (IBZ), ETH Zürich, ETH-Zentrum CHN, Universitätstrasse 16, CH-8092 Zürich, Switzerland

**Keywords:** Sociality, Transmission, Genetics, Generalized distance, Parasite fitness, Social immunity

## Abstract

Parasites and their social hosts form many different relationships. But what kind of selection regimes are important? A look at the parameters that determine fitness of the two parties suggests that social hosts differ from solitary ones primarily in the structure of transmission pathways. Because transmission is, both, the physical encounter of a new host and infecting it, several different elements determine parasite transmission success. These include spatial distance, genetic distance, or the temporal and ecological niche overlaps. Combing these elements into a ‘generalized transmission distance’ that determines parasite fitness aids in the identification of the critical steps. For example, short-distance transmission to genetically similar hosts within the social group is the most frequent process under sociality. Therefore, spatio-genetical distances are the main driver of parasite fitness. Vice versa, the generalized distance identifies the critical host defences. In this case, host defences should be primarily selected to defend against the within-group spread of an infection, especially among closely related group members.

The current SARS-CoV-2 pandemic has made tragically clear what enormous effects parasites can have on social host species. At the same time, it has become obvious that social hosts are under strong selection to defend themselves against these ever-present threats. For human hosts in the current pandemic, this means the need to develop vaccines or to reduce social contacts, such as by social distancing and travel bans. Clearly, group-living and social relationships are important in creating opportunities for parasites to spread among hosts. How sociality dictates this spread and the patterns and processes in which parasites and hosts evolve antagonistically under these amplifying conditions remain important questions and challenging research tasks.

For once, this is not a topic where one can, in passing, refer to early contributions by Darwin himself (Schmid-Hempel 2009), but instead it began to emerge in earnest only four decades ago (Freeland 1976, 1979). A renewed interest in this topic and a more comprehensive scrutiny on the links between host sociality and parasites then focused on social insects (Schmid-Hempel 1998) and primates (Nunn and Altizer 2006). Questions such as the effects of social group size (Patterson and Ruckstuhl 2013), defence strategies of the social hosts (Cremer et al. 2007), health-related costs and benefits (Kappeler et al. 2015), the evolution of parasite virulence in social systems, the effects of parasitism on social evolution, or modelling parasite effects on social groups (Fefferman et al. 2007) have dominated this discussion (Schmid-Hempel 2017). Enormous progress has thereby been made, improving our understanding of social host-parasite systems. But many open questions remain, which is not surprising given the large diversity of social systems, of parasites, and of their interactions. Here, I argue that, collectively, the available studies suggest that—for an infecting parasite—individuals within a social group are not much different from their solitary counterparts. But social hosts dictate a different structure of transmission pathways. A sharpened focus on this crucial difference will therefore be most useful for scrutinizing the elements that determine the success of parasites and their social hosts, as exploring this relationship remains a major empirical challenge.

## The social niche

**Fig. 1:**
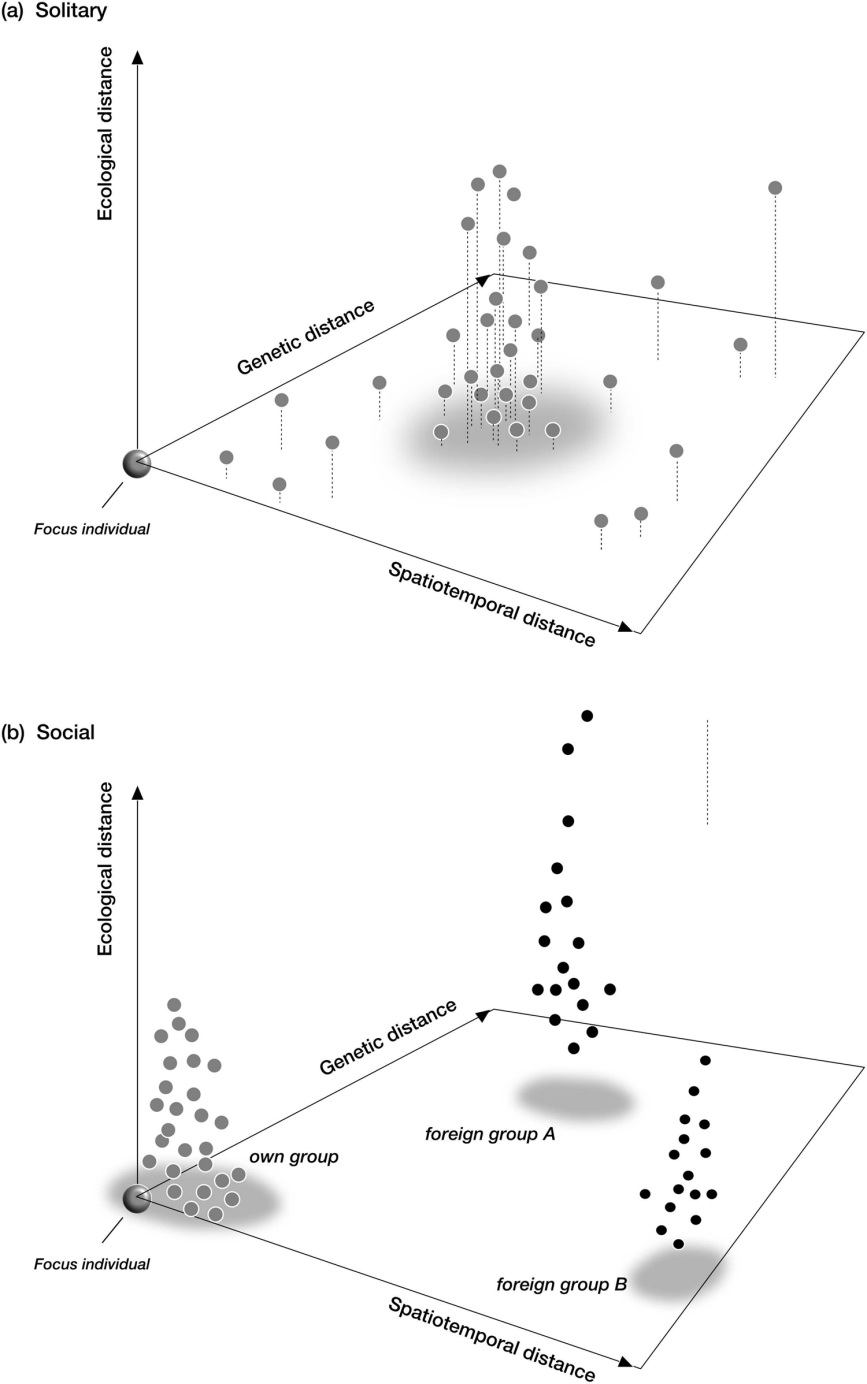
Transmission distances as viewed from a parasite that has infected a focus individual. Elements of transmission distance are spatial and temporal separation to a next host (spatiotemporal distance), ecological separation by host niches (ecological distance), and genetic similarity (genetic distance). The latter results from ‘transmission’ being the encounter *and* the infection of a next host. Dots refer to the position of potential next host in this transmission distance space. **a** Situation in solitary hosts. Most potential new hosts cluster around a mean distance from the focus individual. **b** In social hosts, most potential new host are nearby in this space, i.e. belong to the same social group. Distances to other social groups are much larger. Note that ecological distances may vary more than in social hosts because sociality typically allows to occupy wider niches

Sociality is a common and successful lifestyle with a range of consequences for the organisms and their biotic and abiotic environment. ‘Sociality’ comes in many forms, from a simple aggregation where individuals temporarily gather at a locality for advantages like communal hibernation to the highly evolved eusociality of insects, with reproductive division of labour and task partitioning, and to the even more extreme lifestyle of animals such as the siphonophores where individuals are also physically fused into a ‘higher-order’ organism that acts like a super-individual. In fact, social species vary enormously in many of their characteristics, but—I here argue—opportunity for transmission is the essential difference for parasites infecting a social rather than a solitary species. Therefore, if one were to extract some major axes from what is a multi-dimensional ‘niche’ of social species, four relevant elements will emerge—the temporal, spatial, genetic, and ecological proximities associated with social life and social organization (Fig. 1).

Social insects, for example, form annual or perennial colonies. Examples of the former are bumblebees or wasps, e.g. *Polistes*, and hornets (*Vespa crabro*). Colonies of honeybees, of stingless bees (Meliponini), and those of all ants or termites persist for years, sometimes decades, even though a succession of queens (and kings) within the colony occurs (Boomsma et al. 2005; Starr 2021). At the same time, the spatial distribution of individuals within and among social groups varies considerably. On the one hand, spatially loose aggregations characterize grazing animals such as wildebeest or reindeer that follow temporarily favourable food supplies. Such herds may additionally be structured along families or mating groups. Other social species gather at night in protected sleeping sites but will disperse and forage during the day, such as seen in bats or monkeys. Day activities may occur in small bands and family groups, e.g. in baboons (Schreier and Swedell 2012). By contrast, advanced sociality is associated with persistently close spatial proximity of individuals within social groups, as illustrated by ant colonies or colonies of mole rats. At the same time, close proximity within social groups begets larger distances between groups in several dimensions (Fig. 1). These conditions will clearly affect the transmission of parasites and how infections can spread in social groups.

Ignoring simple social organization levels, such as swarm formation or hibernation aggregations, sociality is furthermore—even though not inevitably—associated with genetic similarities among group members. Hence, individuals within groups are, on average, closer related with one another than with individuals of other groups. Examples are social mammals where groups are formed by family members, e.g. as in marmots, prairie dogs, and monkeys (Altizer et al. 2003; Nunn and Altizer 2006), or in social insects, such as ants, social bees, and most social wasps (Breed 2014). Finally, organizational resilience and cooperation allows social species to expand their ecological niche as compared to solitary species. Examples are a broader spectrum of prey that can be subdued by socially cooperating predators, as known for the large African carnivores (Clements et al. 2014). Similarly, colonies of social insects can regulate their own ambient settings and withstand more adverse conditions as compared to a solitary individual—capacities that have added to the ecological success of social species (Wilson and Hölldobler 2004). Whereas wider ecological niches will not much affect within-group spatial or genetic proximities, it can lead to more contacts with potential pathogens, and niche breadth can even be selected by parasite pressure (Britton and Andreou 2016). Moreover, niche width can also include the temporal patterns of activity. If activity times are longer, this, too, can lead to more contacts with neighbours or reservoirs of novel pathogens. Finally, reproductive output, i.e. how many offspring are produced by a social group or an individual within a group, varies considerably (Jandt and Gordon 2016). There is reproductive skew within societies, such that few individuals contribute disproportionally much to offspring (Nonacs and Hager 2011; Ross et al. 2020). Examples are the dominant males and females in mammalian societies, or reproduction by queens in ants or bees, especially when workers are physiologically sterile or suppressed. Variation in reproductive output will therefore also affect the spread of parasites along with their infected hosts.

One can now ask what section of the multi-dimensional ‘niche space’ of Fig. 1 is particularly favourable to parasite transmission? Importantly, ‘transmission’ includes the encountering *and* the successful infection of a next host. Only if the parasite actually establishes an incipient infection will transmission be complete; otherwise, it will just have physically reached a new host but with no consequence for its spread. Transmission is therefore not only a function of physical, spatial distance among hosts but also a function of how similar the next host is to the current one (which, after all, had been successfully infected). A crucial element of this ‘similarity’ is genotypic similarity, since infection success depends, to a large degree, on genetic factors. Hence, besides close spatial proximity, genotypic proximity among hosts is the second major feature of the favourable corner of the niche space. Incidentally, combining spatiotemporal and genetic proximities for parasite success overlaps with the concept of the ‘ecological filter’ vs the ‘physiological filter’ proposed by Combes (2001). Of course, proximities are also modulated by the changing environmental conditions that affect the transmission process. Moreover, physical proximity depends on transmission mode, too, e.g. whether it is by direct contact, via air, or by vectors.

With these elements in mind, we might view social hosts as being commonly confined within a section of the larger spatiotemporal-genetic niche space that happens to be favourable for parasite transmission (Fig. 1). As argued above, social species are often densely packed as individuals, genetically closely related, relatively stable over space and time, and can even moderate some of the environmental conditions by shaping their living environment. What exactly defines this social corner of the niche has been subject to an enormous research effort over the last decades. This has also clarified the principles of why and along which routes sociality has evolved (Wilson and Hölldobler 2005; Bourke 2019; Araya-Ajoy et al. 2020). But many of the features that favour cooperation and social evolution more generally, such as limited dispersal opportunities, shelter, and nesting opportunities, in fact also facilitate parasite transmission.

**Fig. 2:**
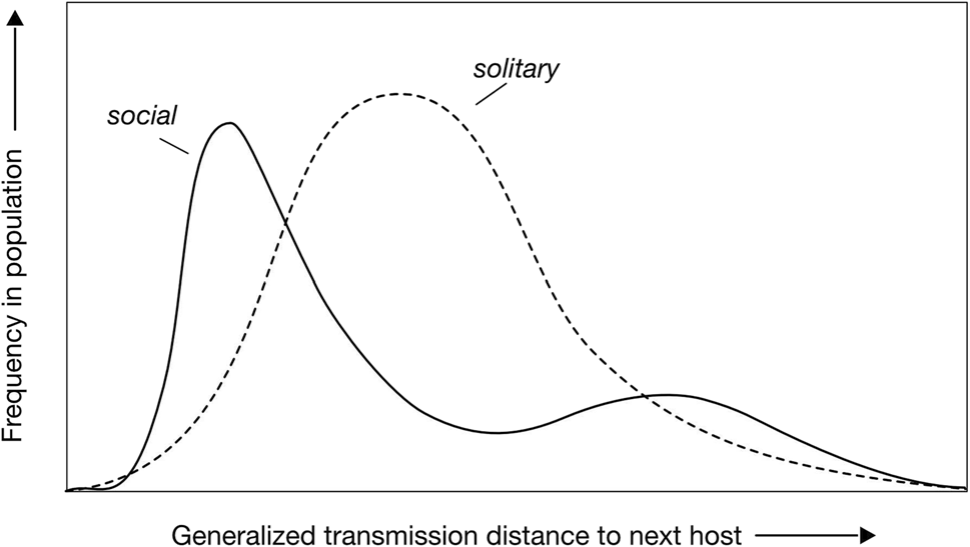
The relevant distance space of Fig. 1 can be captured in a combined ‘generalized transmission distance’ that contributes most to parasite fitness. The distribution of this distance shows a unimodal mean and variance for solitary host, but a bimodal distribution for social hosts. Short generalized distances are the most frequent transmission opportunities under sociality. In addition, the generalized transmission distance in social hosts is mainly set by spatial and genetic proximity

At this point, one can translate the niche space occupied by hosts from the parasite’s perspective into transmission distances of a kind. It is useful to think of a ‘generalized transmission distance’ that combines the mentioned elements, in particular, physical-spatial distance (a function of host density) and genetic distance (given by genetic similarity in the relevant genes). With a generalized distance, and with the rendering of Fig. 2, solitary organisms represent a host space where the generalized distances to a next host are unimodally distributed, with a mean distance that governs the transmissions. Social organisms, by contrast, will assume a bimodal distribution, with hosts from a given social group being close to one another, and the distance to hosts in another social group being large. The rendering of Fig. 2 may look simplistic, but it is a helpful tool for thought because it breaks down the complexities of social organization into manageable components of prime importance for host-parasite interactions and their outcomes. Recall that, by definition, host and parasites are antagonistic in their effects on the fitness of the other party. Therefore, elements that favour parasite fitness, in general, reduce host fitness and vice versa. It is therefore useful to look at factors that affect the fitness of parasites and their hosts before returning to transmission distances.

## Parasite fitness components

Parasite fitness is a product of within-host and between-host components. Within-host fitness determinants result from successful establishment, multiplication (or individual growth as, for example, in helminths), and the eventual production of specialized transmission stages or any other propagules. Between-host fitness results from the ability of the parasite to reach and infect a next host. For both components, we can identify a number of factors that affect success and ask how these differ between solitary and social organisms.

### Within-host success

The within-host success of a parasite results from various processes (Table 1). For the sake of argument, I will focus on three of those. Firstly, once the parasite has managed to infect, resistance (i.e. reducing infection load or clearance of the parasite) and tolerance (coping with the adverse effects of infection, Medzhitov et al. 2012) are two major strategies of individual host defence. These strategies occur on the physiological individual level in both solitary and social organisms. But social groups have additional opportunities for defence, for example, via demographic level adaptations that have effects on parasite within-host growth, too. For instance, social groups may show resilience against the loss of group members that have become infected. As a consequence, individual, within-host defences can be lower (and thereby costs are saved), but this likely will increase parasite success within a host. Resilience against loss is a kind of tolerance strategy at the group level, just as individuals may tolerate an infection. As a defence strategy, this part of the collective host defences, discussed below.

**Table 1:** Fitness components of hosts and parasites

Within-host	Between-host
From the parasite perspective:	
Host resistance and tolerance	Leaving the host
Host stability:	Entering a host
life span, body size, condition	
Competition:	Transmission:
multiple infection by several parasites	within same colony
escape variants	to a next colony
	Host manipulation
From the host perspective:	
Individual immune defences	Avoid infection of colony ('fortress'):
Dispensable individuals (small effect on colony)	Dispensable individuals (remove infection)
Individual tolerance	Behavioural defence within colony: avoidance, discrimination, signalling
Counteract manipulation	Structural defence within colony:
	compartments
	roles
	division of labour

Regardless of the actual defence strategy, and regardless of whether this occurs early or late during the infection process (Duneau et al. 2017), individual defences may potentially differ between social and solitary organisms and therefore differentially affect parasite success. For example, differences in gene repertoires and genomic organization may exist. However, the observed depauperate repertoires of immune genes seem to precede the evolution towards sociality (Barribeau et al. 2015) and are not characteristic of sociality per se, as in the examples of ants (where all known species are social) and social bees (Evans et al. 2006; Gadau et al. 2012; Simola et al. 2013). On the other hand, due to the consequences of social complexity (e.g. as selection acts on workers rather than the queen), organizational resilience (e.g. selective worker death may not significantly reduce queen success), or smaller effective population sizes (e.g. due to large variation in colony reproductive outputs), the strength of negative selection on the genome—which is harboured and transmitted by the sexuals—is reduced by sociality (Imrit et al. 2020). This may help to explain why, for example, the extant genomes of social insects show an expansion and diversification of regulatory genetic elements, whereas (structural) protein evolution seems otherwise more constrained as compared to their solitary counterparts (Simola et al. 2013; Kapheim et al. 2015). It remains to be clarified to what extent these differences affect within-host parasite success in social species, for example, whether the regulation of defences is more frequent and sophisticated with sociality. At least for some cases such as cooperatively breeding birds (Spottiswoode 2008) or social bees (Stow et al. 2007), the expressed individual defences seem stronger in social than in solitary organism. Individual rank within the society can also affect expression of immune genes (Tung et al. 2012), but, at least for males, there seems to be no consistent association across studies in vertebrates (Habig and Archie 2015). Hence, within-host success of parasites in solitary hosts seem not to hinge on very different processes as compared to social hosts.

Secondly, host ‘predictability’ generally favours the persistence and success of an infecting parasite; it should also lead to higher host specialization by the parasite (Combes 2001). Predictability is high when hosts live longer, have larger body sizes, are in good condition, or are simply more abundant. In all of these cases, it is likely that the individual host offers more and more stable resources. Fitting this idea, haemosporidian parasites and fleas are more host-specialized in fish, birds, and mammals with larger body mass or in more abundant host species (Ŝimková et al. 2006; Svensson-Coelho et al. 2016). Individual host condition should be more stable in social hosts, because sociality buffers against fluctuations in available resources. Increased longevity is observed for high-ranking individuals in social mammals, and for queens in social insects and mole rats. More generally, however, there is no consistent increase in longevity with an increasing degree of sociality (Toth et al. 2016; Ellis et al. 2019; Lucas and Keller 2020). As far as host predictability is concerned, social hosts will thus provide a locally consistent and somewhat more predictable resource, which will favour a degree of specialization on the current host. This in turn reduces the generalized within-group distance at the expense of the between-group distance in Fig. 2.

Thirdly, within-host competition with other parasites or variants of the same parasite can reduce parasite success. Such multiple infections or mixed-genotype infections (by the same parasites) are very common in all host-parasite systems, e.g. Balmer and Tanner (2011). Multiple infections are perhaps more common in social hosts than in their solitary counterparts. This is because—and for reasons mentioned above—variants of existing infecting parasites will also find more favourable conditions within the same corner of the social niche. Epidemiologically, also the force of infection (the probability to contract a parasite from an infected individual) is higher with sociality once a parasite is spreading inside a social group. Note that multiple infections (additionally) result from ‘escape mutants’ that emerge within a host individual. Escape mutants emerge when infecting parasite populations can evolve within a host and when selection is imposed by host defences. This happens in the SARS-CoV-2 pandemic where mutations in the spike proteins of some viral mutants escape antibody recognition and thus raise concerns over potential infections of vaccinated hosts or re-infection of patients recovered from a previous infection (Altmann et al. 2021; Greaney et al. 2021). Unfortunately, there is no systematic knowledge on differences between solitary and social hosts with respect to within-host competition frequencies.

Taken together, the individuals belonging to a social group have characteristics that are perhaps marginally different from those a parasite would encounter when infecting a host belonging to a solitary species. More pronounced differences may often result from which individual of a social group is infected—with high-ranking hosts offering more resources and persistence, but probably being more refractory than low-ranking individuals. As a result, the ensemble of parasites infecting the different classes of individuals may differ as in the example of honeybee queens and her workers (Kevill et al. 2020). But from the parasite’s perspective, it matters whether the mean and variance in the defences (in strength, in scope, in repertoire, or in the level of tolerance) among individual hosts is larger in social than in solitary hosts. The answer probably is that it may not differ much. A much-needed systematic survey would clarify these issues. For the time being, we conclude that the within-host success of a parasite infecting a social host does not depend on very different processes than what is encountered when infecting a solitary host.

### Between-host success

This component of parasite fitness covers the steps from leaving the current host to encountering and infecting a next host. Whereas leaving a current social host is unlikely to be different from leaving a solitary host, several other elements are obviously affected by sociality and vary with the conditions in the social niche space. The contribution of genetic proximity has already been mentioned. Beyond this, transmission to a next host can be vertical (i.e. transmission to offspring of the current host), or horizontal, that is, to hosts that are not offspring of the current host. Some parasites of social insects, for instance, can directly be vertically transmitted, such as microsporidia in honeybees through the seminal fluids of males (Roberts et al. 2015). But whereas the distinction of horizontal vs vertical transmission route is important, it can distract from more relevant classifications of transmission in social hosts (Schmid-Hempel 2017). For example, a parasite passed on by the founding queen to her workers in a social insect colony, formally, is vertically transmitted. When the same parasite spreads among workers, formally, this happens horizontally—just as when workers subsequently infect a daughter queen within the colony. Although no direct infection from the queen mother to her daughter queens has happened in this case, this would formally be a vertical transmission once again.

As the example of social insects shows, ‘social transmission’ within groups combines elements of both routes, but these become decoupled from their consequences classically ascribed to either vertical or horizontal transmission. For example, vertical transmission more likely (yet not universally) leads to the evolution of lower parasite virulence, e.g. Ebert (2013). This may still be the outcome for the ‘detoured’ vertical transmission from mother queen to daughter queen via the workers, but the process is not any longer so clear-cut. Similarly, when workers become infected by their mother, this is a dead end for obligately vertically transmitted parasites because workers rarely have offspring of their own or even are physiologically sterile. The same is true when workers do not pass on the parasite to sexual offspring (daughter queens and drones), or when drones infected in this way do not transmit the parasite during mating (i.e. with no sexual transmission). With a macroscopic perspective, vertical transmission will then depend only on the infection of daughter queens in some other way; incidentally, daughter queens also happen to be the least frequent class of individuals within a social insect colony. Similar considerations would in fact apply to all social groups with a substantial reproductive skew, i.e. with a very unequal distribution of reproductive success among members of a social group (Keller and Reeve 1994). In fact, with the basic rendering of Fig. 2, a generalized transmission distance appears to be more useful for capturing these situations. Within social groups, parasites transmit to close neighbours in this generalized distance space, for example, to a worker or a daughter queen in a colony of social insects (intra-colony infections). Occasionally, transmission is over longer distances to a host in another social group (inter-colony infections). Whereas long-distance transmission is almost always horizontal, a distinction to horizontal transmission is not always helpful for close-distance events. In any case though, the structure of transmission distances is massively affected by sociality and its precise organization. Therefore, the between-host success of parasites in social host deviates from the between-host success in a solitary counterpart. Parasite transmission in structured populations is, of course, not an entirely new subject and has been discussed for some time (Haraguchi and Sasaki 2000; Kamo et al. 2007; Tellier and Brown 2011). Long-distance transmission is also a consequence of migration—where individuals move between different areas on a regular basis, and of dispersal, i.e. offspring that leave the natal area. An example is protozoan infections of monarch butterflies (*Danaus plexippus*) that gather in large numbers in overwintering sites in Mexico. These infections are carried by the migrating individuals but are successively ‘left behind’ in the process as the infection weakens its carriers (Bartel et al. 2011; Altizer et al. 2015). Similarly, regular nest movements by social insect colonies may be forced by the presence of parasites (McGlynn 2011). Migration strategies can therefore lower long-term parasite success. Similar consequences can result from dispersal strategies (Boulinier et al. 2016), including the effects of spillovers to resident host populations.

## Host fitness components

The cascade of defences against parasites starts well before an infection actually occurs. This includes choice of a microhabitat, avoidance of risky places, daily routines, staying away from infected group members, and so forth (Parker et al. 2011; de Roode and Lefèvre 2012; Schmid-Hempel 2021). In response to infection risks, strategies of prophylaxis are known whereby individuals up-regulate their immune defences (Wilson et al. 2002), collect protective chemicals (Huffman 2016), or change their behaviour (Feener 1988). Only after infection has become unavoidable will individual defences be deployed.

### Individual defences

Individuals can reduce the risk of infection by a changed behaviour, and also somewhat independently of the social group, for example, when risky places or a risky diet are avoided (Curtis 2014). Whether sociality reduces or augments these individual pre-infection defences is not clear. With an infection, physiological defences—notably, the immune defences—are activated. There is evidence that such individual, physiological defences covary with sociality, yet not necessarily in a consistent manner. Indeed, social species may show increased individual defences (Stow et al. 2007; Hoggard et al. 2011; Turnbull et al. 2011), but the reverse is also observed (Wilson et al. 2003). A reduced defence could also result from individuals being ‘dispensable’ for the social group (see below).

A particularly important phenomenon is individual protection against infection by some kind of immune memory (in its widest sense). ‘Within-individual’ protection provides increased resistance to a second infection by the same or a similar parasite. This is classically the case with the ‘immune memory’ of the jawed vertebrates that is based on B- and T-lymphocytes. The same phenomenon is observed in invertebrates, too (Sadd and Schmid-Hempel 2006; Milutinović and Kurtz 2016), and is commonly known as ‘immune priming’ to emphasize—compared to vertebrates—the different scope and molecular mechanisms involved (Little and Kraaijeveld 2004; Pradeu and du Pasquier 2018). A second important category is the protection of offspring against the same or similar infections by their parents, via some kind of priming of the offspring’s immune defences (‘trans-generational priming’; see Roitberg and Rosengaus in this Topical Collection; Roth et al. 2018; Tetreau et al. 2019). In mammals, this can happen via compounds transferred by mother milk, but trans-generational priming also occurs in invertebrates, e.g. in mealworms (Moret 2006), moths (Tidbury et al. 2011), or social insects (Sadd et al. 2005), but the mechanisms are as yet poorly understood. Immune memory and priming clearly benefits individual hosts of solitary and social organisms alike, but its fitness consequences are quite different.

### Collective (social) defences

Obviously, sociality offers additional possibilities for defence against parasites. Collective defences, also discussed as ‘social immunity’ (Cremer et al. 2007; Masri and Cremer 2014), are certainly a hallmark of sociality. Examples are simple, hygienic behaviours such as allogrooming, the application of antimicrobial compounds to brood, the removal of infected group members (Meunier 2015), or cannibalism—the killing and ordered disposal of infected colony members to interrupt the infection chain. A further example is gallery construction with faecal material rich in antifungal activity as observed in termites (Rosengaus et al. 2011). Furthermore, the loss of a colony member may not have large effects on the fitness of other members and the group, respectively. In fact, ‘dispensable individuals’ may be particularly important for colonies of social insects where workers forgo reproduction as categorized by Straub et al. (2015; ‘superorganism resilience’), but less when all group members have some perspective for own reproduction. In such cases, within-group conflict over whom should perform the risky tasks (e.g. vigilance against aerial predators as in meerkats) will emerge and needs to be settled, for example, in a context of family groups.

Protection by immune memory or immune priming is not only a part of individual defences but is embedded in the social context and, therefore, also a part of collective defences. In social insects, for example, a signal for priming can be from the mother queen that may also have carried the same infection into her colony and now can protect her offspring by trans-generational immune priming. Furthermore, individuals can additionally be protected by ‘social priming’ as in ants (Matthias et al. 2012) or termites (Traniello et al. 2002), where the signal comes from group members rather than from parents or own experience. Essentially, collective defence against infection in humans also is an example of social priming. Additionally, protection by memory and priming takes effect before every group member is protected individually, that is, when the threshold for herd immunity is reached and the parasite cannot spread further due to a lack of susceptible host individuals. Herd immunity can also be reached in a population of solitary hosts, but the process that leads to this state is likely to be less directed and less efficient. In fact, whereas herd immunity in a social group benefits primarily the group members and thus indirectly adds to the fitness of the immunized individuals themselves, herd immunity in a solitary population benefits all individuals regardless of their relatedness and whether they carry the cost of becoming immune or not. Matched for the same cost, the evolution towards immune priming should therefore be more likely in social hosts.

Finally, defence can be by ‘organizational immunity’. The possible pathways of parasite transmission are thereby constrained by the organization of contacts within the social group (Schmid-Hempel and Schmid-Hempel 1993; Naug and Camazine 2002; Altizer et al. 2003; Pie et al. 2004). An example is the spatially separated nest chambers used by workers of the leaf-cutter ant, *Atta colombica*, that are specialized for waste management, and which thereby reduces contacts with workers attending the valuable fungus gardens (Hart and Ratnieks 2001). Many other examples have been described, mostly in ants and social bees (reviewed in Stroeymeyt et al. (2014)). A related phenomenon is an adaptive shift in task allocation in social insects that compensates for deficiencies caused by killed or incapacitated workers (Natsopoulou et al. 2016). Hence, with organizational immunity, e.g. by physical compartmentalization and task specialization, the spread of a parasite in a social group can be impeded, as in the case with adaptive social organization of the garden ant, *Lasius niger* (Stroeymeyt et al. 2018). How parasites are distributed within a social group can also affect the spread to another social group (Ulrich and Schmid-Hempel 2015). The various mechanisms of collective defences have been discussed intensively over the last decade (Cremer et al. 2007, 2018; Rosengaus et al. 2011; Masri and Cremer 2014; Kappeler et al. 2015). Sociality adds to defences against which the parasite must evolve adaptations, but not every mechanism of collective defences need evolve in any given case (Meunier 2015).

## Consequences for parasites and hosts

By definition, parasites gain fitness at the expense of their hosts and vice versa. But parasites depend more on their hosts than the hosts do on their parasites (the ‘life-dinner principle’, (Dawkins and Krebs 1979)). In addition, most parasites are more numerous and have shorter generation times than their hosts. This asymmetry indicates that, in most cases, parasites are the pacemakers of the co-evolutionary ‘race’ (and this should show in the parasite genome). Hence, where parasites can gain most of their fitness will define the most relevant elements of the host-parasite interaction. Figures 1 and 2 suggest that most between-host transfers are ‘close transmission’ events as many more opportunities arise to transmit within a social group than becoming transmitted to another social group. It is therefore close at hand to suggest that such short-distance transmission events dominate the evolution of the social host-parasite interactions.

Incidentally, close-distance events amount to a serial passage of a parasite through the same or similar hosts, where an experimenter that manually infects a next host bypasses the natural transmission step. With such serial passage experiments, increased parasite specialization and virulence is observed (Ebert 1998). In natural systems, increasing specialization on the current social group can, in the extreme, lead to the evolution of symbiosis, e.g. Hughes et al. (2008). On the other hand, an increasing specialization requires the persistence of the current host lineage over time. This will never be certain. Such uncertainty selects for long-distance transmission that allows to ‘jump’ to another host lineage with some frequency (Ebert 2013). Long-distance transmission is therefore not entirely irrelevant but puts limits, for example, on the evolution towards specialization. For the parasite, strategies of long-distance transmission are akin to dispersal and colonization strategies—with high risks but also high rewards. Relevant for the current discussion, the relative weight of these two distance categories varies with social organization, from ephemeral group living during the breeding season to the long-lasting perennial colonies such as those of ants.

With short-distance transmission plausibly being the most important component of parasite fitness, host defences should focus on this element, too, due to the inherent reciprocity of effects. In other words, host defences that reduce within-group transmission should yield higher fitness gains than any other measure. Furthermore, if this view is correct, preventing a primary infection of a social group in the first place (through long-distance transmission) is not necessarily the primarily selected process. This remains the case despite the fact that keeping out the infection from a social group is enormously efficient to increase health and success of all members of a social group. For any one host-parasite system though, the relative frequencies of these two transmission distance events will combine with their effects on host (and parasite) fitness to eventually shape the evolution of host defence strategies. If, as is argued here, parasites and short distances set the pace, for most cases, the defence against contracting a primary infection from outside the group will nevertheless recruit the same defences that actually have evolved for defence against the within-group spread of an infection.

This also gives the collective defences or social immunity a prime role. Among all within-group defence measures, reducing transmission opportunities by behavioural change is very effective at a low cost. In social groups, therefore, we can conclude that behavioural changes are very effective strategies of defence and are prominent adaptations in social animals. This should include the evolution of cues and the sensory repertoire to identify infections that would then allow for plasticity in behavioural defences (Rosengaus et al. 1999; Richard et al. 2012; Pull et al. 2018). Similarly, immune priming and compartmentalization by organizational immunity are further elements that promise large effects at a moderate cost. Indeed, social organization is also well-captured with contact networks. Across many species of vertebrates or social insects, social contacts vary most in solitary species. Yet, contact networks are most fragmented in gregarious species, testifying to the relevance of organizational immunity (Sah et al. 2018). Organizational immunity is restricted to social hosts, but strategies of immune priming can be found in all organisms. Based on the arguments put forward here, immune priming should be strongly selected in social hosts to defend against the within-group spread of parasites. Theoretical models, e.g. through analyses of evolutionary stable strategies for, both, parasite and hosts, may be a next step to scrutinize the validity of these arguments and a guideline to empirically test assumptions and predictions.

## Transmission mode vs sociality

Transmission mode, such as vector vs airborne transmission, is a parasite trait that can evolve (Antonovics et al. 2017). Not all modes seem to occur equally often, however. For example, there seem to be few parasites in the eusocial insects that are vector-transmitted, although examples such as tracheal mites (*Varroa*) that disseminate viruses (Wilfert et al. 2016) are proof of existence. By contrast, vectored parasitic diseases are common in mammalian or avian social groups, e.g. blood parasites that are vectored by mosquitoes. This difference may simply reflect a lack of data, but it illustrates the basic issue—does social organization determine the transmission mode?

The spatial and genetical distance to a next host is typically not under the control of a parasite—when ignoring parasite-induced manipulations of behaviour that bring hosts into close physical contact. But the effect of distance is certainly intertwined with transmission mode. For instance, when small airborne particles are the major parasite propagules, close proximity is required (transmission by direct contact). Transport by water can bridge larger distances, and vector transmission can reduce spatial separation and the genetic distances by a targeted behaviour of the vector itself. At the same time, spatial-genetic separation from other groups is a defining element of host sociality and varies with the level of sociality. Social organization is thus expected to affect the conditions which favour different transmission modes.

Table 2 is an attempt to structure these patterns. For any category of Table 2, many exceptions could be found. But, for example, direct transmission by close body-to-body contact can happen frequently and is efficient within, but rarely between social groups. By contrast, vectored transmission would be quite efficient to transmit a parasite from one social group to a nearby one. But even though vectoring between groups occurs, the sheer frequency of within-group opportunities may eventually select for the more efficient direct transmission mode at the expense of vectored transmission. In social insects, for instance, it almost seems easier for a pathogen to achieve between-colony transmission by directly infecting the dispersing sexual offspring of a colony, or by exploiting ‘drifting’ workers that erroneously enter foreign colonies, than by being vectored to an unrelated colony. In that case, selection for efficient direct transmission would maximize both within- and between-colony transmission. Furthermore, the sharing of resources will add to the effect, for example, when workers of social bees deposit infective parasite propagules on flowers that are thereby directly transmitted to a next, visiting bee from another colony (Durrer and Schmid-Hempel 1994). Similar arguments may suggest why vectored transmission (or, alternatively, passive transmission by air or water) may instead evolve in swarming fish or in loosely interacting herds of ungulates, since between-group transmission becomes relatively more frequent and vectors or passive transport are an efficient means for this.

**Table 2:** Social organization (with example) and the epidemiology of transmission by different modes. For simplicity only three major modes are mentioned. (Note that these scenarios are for transmission between individuals rather than by direct vertical transmission to offspring, e.g. via eggs. Also note that the scenarios do not explicitly consider life cycles with several different hosts, e.g. dixenous trematodes that can infect ants)

	Within-group opportunities			Between- group opportunities		
Degree of sociality and characteristics	Direct by contact	Passive by air or water	Vector active search	Direct by contact	Passive by air or water	Vector active search
Solitary	Rare: individuals are dispersed and meet rarely	Moderate to common: in relation to distances	Moderate to common: can target other individuals even if distant	–	–	–
Swarm, aggregation: ephemeral groups of random individuals (bird flocks, fish swarms)	Very rare: individuals are not interacting closely	Rare to moderate: individuals are not always close but can be numerous	Moderate: individuals are not dense but can be targeted by vector	Moderate: other groups distant, but individuals of different swarms mingle	Moderate: other groups are reasonably close	Moderate: other groups are reasonably close
Colonial: periodically existing, or more persistent groups of individuals (colonially nesting birds, breeding seals)	Rare: individuals remain separate, with occasional contact	Moderate: individuals remain separated or are only present for a limited time	Moderate to common: individuals can be targeted by vector	Rare: other groups far away and typically exclusive (xenophobic)	Rare to moderate: other groups are not close and may not be reached by passive transport	Rare: other groups not close
Family groups: persistent groups based on related individuals (herds of ungulates, sea mammals)	Moderate to common: family group members interact closely but groups small in numbers	Moderate to common: contact close but limited in numbers	Common: vector can target close individual of similar type, but limited numbers	Rare to moderate: other groups away and typically closed for outsiders	Moderate to common: other groups are in vicinity or come close occasionally	Moderate to common: other groups in vicinity and can be targeted
Sociality (eusociality): persistent, dense, and typically closely related individuals (social insects, mole rats)	Very common: individuals interact closely and groups typically numerous	Very common: close and frequent contacts among members	Very common: vector can immediately reach many close individuals of similar type	Rare: contacts between groups very restricted	Moderate: other groups are some distance away and separated	Moderate to common: other groups are some distance away, but can be targeted
Extreme sociality: individuals form complex organisms by fusion of their bodies (siphonophores, some porpitids, and bryozoa)	Inevitable contacts among individuals	Persistent opportunities	Persistent opportunities	Very rare: contacts between groups extremely rare and separation defended	Moderate: other groups are close, or up to some distance away, but often rare in habitat	Moderate to common: other groups are close, or up to some distance away

But as social organization becomes more complex and more tightly integrates the group members, selection on transmission within the group increases relative to transmission to other groups—and this likely selects for direct transmission strategies. Obviously, this also depends on how quickly all available hosts within a group have already become infected and thus become potentially unavailable, such that the transmission to a next group becomes favoured again. This point will take longer to reach in larger social groups, when no lasting primed immunity exists. Similarly, when, by births, new and naïve group members are recruited into the society frequently enough, there will always be enough new hosts to infect, and the within-group transmission can continue (Büchel and Schmid-Hempel 2016). Finally, if new variants appear in the parasite population at a sufficiently high rate, within-group transmission will also continue. In any case, the spread of the parasite is more likely to stop when it has a relatively short serial time (i.e. the time from infection to infection) and can rapidly spread (Naug and Smith 2007).

## Concluding remarks

This contribution raises some general points on the critical elements in the interaction between parasites and their social hosts. It suggests that the major difference between solitary and social hosts is not in the defences by individual hosts and the within-host parasite success, but in the transmission pathways. A ‘generalized transmission distance’ can capture the most important factors that determine parasite success and, therefore, the most critical defence elements of social hosts. In particular, scrutinizing the generalized transmission distance suggests that within-group transmission is the most important selective episode for the evolution of social hosts and their parasites. These effects can be modelled (Fefferman et al. 2007; Hock and Fefferman 2012; Sah et al. 2018; Guo et al. 2020), and sensitivity analysis (e.g. sensu Frank and Schmid-Hempel 2008) will provide further guidelines as to which elements of the generalized distance are most critical for either party. In addition, there is as yet only limited empirical evidence for most of the potentially crucial processes, which therefore remains a major task. Future research might scrutinize the possible elements of the generalized transmission distance and to identify which of those will be the most crucial ones in different social systems. This may help to understand what trajectories social evolution has taken through the space covered by transmission distances.
